# An assessment of client and clinician satisfaction in veterinary teleconsultation compared to in-person consultations

**DOI:** 10.18849/ve.v7i3.578

**Published:** 2022-02-27

**Authors:** Narakhanti Soenardi+, Maxim Bembinov+

**Keywords:** CLIENT, CLINICIAN, CONSULTATION, PRACTICE MANAGEMENT, REMOTE CONSULTATION, SATISFACTION, TELECONSULTATION, VETERINARY

## Abstract

PICO question

Compared to in-person veterinary consultations, does teleconsultation lead to similar levels of client and clinician satisfaction?

Clinical bottom line

Category of research question

Qualitative assessment

The number and type of study designs reviewed

Eight studies were critically appraised. There were six cross-sectional studies, one randomised controlled clinical trial, and one case report

Strength of evidence

Weak

Outcomes reported

All eight studies provided weak evidence of similar levels of clinician and / or client satisfaction

Conclusion

Teleconsultation can lead to similar levels of client and clinician satisfaction when compared to in-person consultations. However, the evidence is weak due to the subjectivity and varied methods of measuring satisfaction. Furthermore, the current applicability of veterinary teleconsultation is still very limited to certain select scenarios in which it is appropriate (e.g., emergency, triage, remote locations, non-complicated routine postoperative checks, nutrition and behavioural consults)

How to apply this evidence in practice

The application of evidence into practice should take into account multiple factors, not limited to: individual clinical expertise, patient’s circumstances and owners’ values, country, location or clinic where you work, the individual case in front of you, the availability of therapies and resources.

Knowledge Summaries are a resource to help reinforce or inform decision making. They do not override the responsibility or judgement of the practitioner to do what is best for the animal in their care.

## Clinical scenario

Since the onset of the COVID-19 pandemic and the introduction of the various restrictions, many regulators have been forced to reconsider their stances on telemedicine. The Royal College of Veterinary Surgeons (RCVS) had even temporarily allowed remote prescription in absence of any other option (RCVS, 2020). Portugal and many other European countries had similar measures (Magalhães-Sant’Anna et al., 2020) and the US Food and Drug Administration also eased their limits on veterinary telemedicine (FDA, 2020). The French government has gone even further and in May 2020 started an 18 month experimental trial of telemedicine (Legifrance.gouv.fr, 2020). These developments along with growing technological capabilities and consumer interest make the following scenario more and more likely.

You are a practice manager who is considering offering your clients a new service of telemedicine, specifically teleconsultation. Several benefits come to your mind such as better COVID-19 safety, reduced travel and wait time for the clients, etc. However, before you try offering remote consults you wonder if there is an evidence base to find out if your clients and your clinicians would be satisfied with this service to the same extent they are satisfied with the in-person consults.

## The evidence

Eight studies were critically appraised in this Knowledge Summary, including six cross-sectional studies, one randomised controlled clinical trial, and one case report. Most studies looked at opinions of clinicians and clients involving both quantitative and qualitative data, through the means of surveys and interviews.

Of the six cross-sectional studies, one was exclusively qualitative (Butler et al., 2021), and the rest were quantitative or a combination of both. Together they provided a comprehensive overview of their participants’ outlook towards teleconsultation. The paper with the strongest design was by Bishop et al. (2018) which involved a randomised clinical trial comparing levels of client satisfaction using teleconsultation versus that of a routine in-person postoperative consultation. The case report by Donham & Wickett (2018) showed how teleconsultation can be useful in a very remote location where veterinary care is not available, although it does not directly answer the PICO question.

From all the papers, there is a consensus that teleconsultation can provide a similar level of satisfaction when compared to in-person consultations. However, most studies performed looked at very specific situations or demographics and hence the applicability of teleconsultations in other scenarios would be difficult, if not impossible, to assess. Overall, the biggest issue with the studies appraised was the fact that most studies (with the exception of Griesel, 2017 and Bishop et al., 2018) did not directly measure ‘satisfaction’, thus in order to infer satisfaction levels, other phrasings and proxy measures had to be used. Furthermore, most of the studies did not have a large enough sample size to confidently draw generalised conclusions.

## Summary of the evidence

**Bishop et al. (2018):** 

Population:	Client-owned dogs who have undergone elective surgical sterilisation and post-surgical recheck examination at the Coastal Animal Hospital in Encinitas, California, between September 27, 2017 and February 23, 2018.
Sample size:	30 owners.
Intervention details:	Dogs that were eligible (no complications or other factors that necessitated in-clinic examinations) were randomly assigned to have their recheck examination performed remotely through a videoconferencing application (teleconsultation group – 17 dogs) or in-person at the veterinary clinic (control group – 13 dogs). Owners completed a survey following the recheck examination regarding their satisfaction with the exam and their dogs’ behaviour during it.
Study design:	Randomised controlled clinical trial.
Outcome studied:	Subjective assessments of: Owner level of satisfaction with the recheck examination. Typical time spent travelling to the clinic. Owner perception of quality of service provided during video-conferencing (teleconsultation group only). Owner willingness to use teleconsultation in the future (teleconsultation group only). Responses to ordinal questions were rated on a Likert scale of 1 (strongly agree) to 5 (strongly disagree). Mann-Whitney U test was used to assess if the two groups had a statistically significant difference.
Main Findings (relevant to PICO question)	Clients were equally satisfied with recheck examination performed remotely and in the clinic. Mean ± standard deviation (SD) scores for owners’ responses to perception of veterinarians’ ability to assess postoperative care during the recheck examination were 1.06 ± 0.24 for the teleconsultation group and 1.00 ± 0.00 for the control group but differences were not significant (P = 0.382). Based on the Likert scale questions, owners in the teleconsultation group responded as having been less inconvenienced by their appointment (mean ± SD = 4.17 ± 0.57) than owners in the control group (mean ± SD = 3.85 ± 1.46) but differences were not significant (P = 0.17). Median time for a round-trip travel to the veterinary clinic was 50 minutes (range: 10–60 minutes). Owners with dogs in the teleconsultation group (mean ± SD = 4.76 ± 0.55) indicated that ‘their dogs were less afraid or nervous during the examination’ than the owners in the control group (mean ± SD = 3.85 ± 1.51) but the difference did not show to be statistically significant. All 17 owners agreed or strongly agreed that they felt comfortable using the technology, and that they would agree to have another follow-up visit through the teleconsultation application. 15/17 owners agreed or strongly agreed that the visual and sound quality of the videoconference was clear. 14/17 owners agreed or strongly agreed that they thought their dog were more comfortable than it would have been in the veterinary clinic.
Limitations:	Small sample size due to duration of study and selection criteria of both dogs and owners. Impossible to blind clients and clinicians to interventions, hence may have biased results of the survey. Study was carried out in one hospital and hence the technology and other features of the hospital might not be applicable to other establishments. Travel time reported might be longer than the average client of the hospital as the hospital receives many referrals from other clinics for laparoscopic ovariectomy (inclusion criteria for female dogs in this study). Study might have suffered from positive (positive satisfaction bias) since respondents are more likely to give positive answers when directly asked if they are satisfied (Choi & Pak, 2005).

**Bishop et al. (2021):** 

Population:	Veterinarian members of the Veterinary Information Network (VIN) working in small animal general practice in North America.
Sample size:	550 veterinarians.
Intervention details:	Anonymous online survey consisting of 20 to 28 questions assessing use and insight into synchronous video-based teleconsultation. Survey was sent to 69,488 recipients, 680 responded, and 550 of them reported to work in small animal general practice and had North American internet protocol addresses. Response collected between September 28 and October 21, 2020.
Study design:	Cross-sectional study.
Outcome studied:	Respondents who reported use of synchronous video-based teleconsultation were asked about frequency of use, percentage of participating clients, time required, financial compensation, impact on client relationships, ease of adaptation, and intentions for its use once COVID-19 restrictions are lifted.
Main Findings (relevant to PICO question)	Teleconsultations lead to similar levels of satisfaction compared to in-person consultations with it being more suited for some types of consultations than others. Most clinicians would use teleconsultation less, except for the same or increased level of use with postsurgical care (23/51) and nutrition consultations (12/24). From the 135 respondents who started using synchronous video-based teleconsultation and who answered the questions: 98/135 (72.6%) indicated that teleconsultation took either the same or less amount of time as in-person consultations. 86/130 (66.2%) clinicians reported little to no difficulty in adapting to videoconferencing. All 135 found it was somewhat more difficult to foster a good client relationship and to convey information. 103/135 (76.3%) reported somewhat less or much less financial compensation.
Limitations:	Teleconsultation was defined only as synchronous videoconferencing with clients, and not other forms e.g., phone, email, texts. Study has a low response rate 680/69,488 (1.0%) which could lead to bias towards clinicians who had a positive experience with, or a keen interest in teleconsultation.

**Butler et al. (2021):** 

Population:	UK racehorse trainers and racehorse veterinarians.
Sample size:	10 trainers (four female and six male) and 10 veterinarians (all male).
Intervention details:	Veterinarians and trainers were found through snowball sampling and qualitative semi-structured interviews were carried out between September 2020 and January 2021.
Study design:	Cross-sectional (qualitative) study.
Outcome studied:	During the interviews the following topics were discussed: ‘trainer–vet relationship (pre COVID-19 pandemic)’ ‘changes in trainer–vet relationship during the first ‘lockdown’’ ‘beneficial changes for racehorse veterinary care’ ‘problematic changes for racehorse veterinary care’ ‘management changes enforced during the first ‘lockdown’’ ‘innovative features of veterinary advice, diagnosis and treatment (veterinarians) and information sources of new veterinary practises (racehorse trainers)'. Thematic analysis of the transcripts of three video conferences and 17 telephone calls obtained during the semi-structured interviews, was carried out. Frameworks of critical realism and social constructionism were combined to carry out the thematic analysis.
Main Findings (relevant to PICO question)	As the use of teleconsultation increased during COVID-19, both the veterinarians and the trainers have found it to be useful to exchange images and videos to triage or deal with simple cases; however, not as universal replacement for in-person consultations. Therefore, the conclusion is that it can provide satisfaction levels similar to those of in-person consults, yet only for select cases when it is appropriate. Some of the criticism was poor connectivity, poor quality of the images and videos. Some trainers were also unhappy to be billed for remote consultations.
Limitations:	While semi-structured interviews have a plethora of benefits, (e.g., facilitating quicker triage of the emergency cases, providing quick advice on simple problems) the absence of quantitative data means that the conclusions rely entirely on the study authors’ subjective analysis of the transcripts. Snowball sampling also meant that there was an inherent bias in the selection of the respondents; however, this is a common issue when a specific small group needs to be sampled. Nonetheless, this means that care needs to be taken when extrapolating the conclusions from this study. The interviewer was not blinded to the hypothesis and the purpose of the research, and thus the study might have suffered from the bias associated with the interviewer consciously or subconsciously selecting data that fits with the existing hypothesis.

**Donham & Wickett (2018):** 

Population:	Critically ill military working dog (MWD) presented to The Canadian North Atlantic Treaty Organization (NATO) Role II facility in Iraq without in-person veterinary support.
Sample size:	1 dog.
Intervention details:	An MWD in a remote outstation was sent to a Canadian Role II facility to be evaluated, as it seemed critically ill and sepsis was a concern. Role II medical providers consulted with the MWD unit’s veterinarian through FaceTime (synchronous video-conferencing application).
Study design:	Case report.
Outcome studied:	Whether the FaceTime call could provide evaluation, treatment, and prioritisation of medical evacuation in this scenario where veterinary care is not immediately available.
Main Findings (relevant to PICO question)	While this case report does not have a commercial client, the client is the NATO military, and since the military was satisfied this can be considered as client satisfaction. FaceTime allowed the unit veterinarian to help coordinate care including initial diagnostics and treatment. However, they were unable to definitively diagnose the disease.
Limitations:	Involves military physicians and personnel who had access to medical resources (e.g., laboratories and imaging facilities) which might not be applicable to the general population. Very specific case presentation and so might not be applicable to other disease processes. Case report is a low level of evidence.

**Dubin et al. (2021):** 

Population:	Small animal veterinarians working in the USA.
Sample size:	93 veterinarians (47 in primary care and 46 in specialty practice).
Intervention details:	An online survey (21 questions) was sent between 15 June and 15 July 2020. All veterinarians offering clinical rotations in small animal medicine for 3^rd^ and 4^th^ year students according to the Western University of Health and Sciences College of Veterinary Medicine database were all sent an invitation to participate in the survey. Questions asked about changes in the use of telehealth, demographics, perception of client’s concerns regarding the transmission of COVID-19 via pets, and changes in caseloads before and after the beginning of the COVID-19 pandemic.
Study design:	Cross-sectional study.
Outcome studied:	Data was reported as proportions and percentages of the total. Several statistical analysis methods were used for the demographics section of the study; however, these findings are outside of the scope of the current Knowledge Summary.
Main Findings (relevant to PICO question)	Dermatology (9/33) and ‘triage or emergency’ (9/33) were by far most often put as consults most frequently done remotely. The ‘most commonly ranked first benefits’ of client-facing teleconsultation were safety 10/21 (48%) followed by positive feedback from clients 8/21 (38%) and efficiency (5/21) (24%). When asked about problems encountered while implementing teleconsultation the overall most common challenges were technology (6/20) and client interest (5/20).
Limitations:	Most of the veterinarians who responded to the survey were from California, which means that the results should not be blindly extrapolated as the study found statistically significant relationships between practises offering telemedical services and various demographic factors. In this study, not all of the respondents have answered all of the questions. So even though the total sample size is 93, respondents could pick and choose which questions to answer leading to some questions having 30 total responses or even lower. This could lead to a selection bias, as those feeling more strongly about teleconsultation are more likely to have responded. Given the high number of questions (21 question in total) and high number of questions left unanswered by some respondents (number of answers to PICO relevant questions ranged between 20 to 33 out of 93 total responses), it is possible that some answers given were affected by response fatigue. As it is more likely that once tired of answering questions a respondent will give all ‘yes’ or all ‘no’ answers towards the end (Choi & Pak, 2005).

**Grisel (2017):** 

Population:	Horse owners and trainers (mainly in the south-eastern USA).
Sample size:	83 owners / trainers.
Intervention details:	Survey was sent to 3,200 trainers and horse owners identified using the practice database of the Atlanta Equine Clinic.
Study design:	Cross-sectional study.
Outcome studied:	The main themes in the survey were about the basic demographic profile, the current extent of video acquisition of lame horses, current utilisation of the Internet to post video footage of lame horses, number of requests of professional review of video footage and petition for future veterinary telemedical services. The responses were processed to obtain percentages and when appropriate means and standard deviations.
Main Findings (relevant to PICO question)	This study found that 59/83 (71.1%) of respondents already used telemedical evaluation of their horse’s lameness. 51/59 (86.4%) were satisfied with the review their horse’s lameness received. 65/81 (78.3%) of total respondents reported that they would prefer veterinarians to offer telemedical reviews as a service. 81/83 (97.6%) of respondents said that they would pay for this service if it was offered.
Limitations:	This is a 2017 study, which means that the sentiments and the data collected in this survey might not be accurate in the current climate, as teleconsultation and its technological capabilities are rapidly changing. Survey had a very low response rate (83/3200) 0.02%. The author has a declared conflict of interest as he is an owner of a company that is involved in remote assessment of lameness. While the authors did not limit their sample to a certain geographical location, most of their respondents were from south-eastern USA due to the location of their clinic. This poses a risk as data might not hold true across other populations.

**Magalhães-Sant’Ana et al. (2020):** 

Population:	Portuguese veterinarians.
Sample size:	41 veterinarians.
Intervention details:	As a part of a larger Policy Delphi Study* the veterinarians, identified through snowball sampling, were sent an online questionnaire over the period between September and December 2018. The questionnaire had four main themes: ‘role of the Ordem dos Médicos Veterinários (OMV), the local veterinary regulatory body in Portugal, in telemedicine’, ‘remote consultations’ and ‘teleconsulting and teleadvice’. The veterinarians were then asked to provide a grade using the 5-point Likert Scale (1 – strongly disagree; 5 – strongly agree), N/A was also always an option. Then veterinarians were asked to write a text explanation for their answer. *Policy Delphi Study is a study method that aims to explore both pro and con arguments of a policy, rather than achieve a consensus. (Linstone & Turoff, 2002).
Study design:	Cross-sectional study (with text explanation being a qualitative addition).
Outcome studied:	Means and standard deviations were calculated for the Likert Scale values, which was then interpreted to quantitatively assess the extent to which the respondents agreed with the statements. Subjective interpretation of the comments provided by the veterinarians to explain their responses was also carried out.
Main Findings (relevant to PICO question)	Overall, the veterinarians had a positive outlook on remote consultations. 21/41(51%) agreed that ‘Video-conference remote consultations can, in certain cases, replace face-to-face consultations.’ 25/41 (61%) agreed that ‘Remote veterinary consultations are an opportunity to improve animal healthcare.’ However, 28/41 (68%) broadly agreed that remote consultations should be preceded by a physical exam. Though, in cases of emergencies (e.g., poisonings or heatstroke) in which the speed of receiving guidance can mean the ‘difference between life and death’; or in behavioural medicine where it could be the only way of achieving an accurate diagnosis (as the animal would be in its normal environment), remote consultations are vital even without a physical exam. One large animal practitioner pointed out that remote consultations had already been a part of farm practice for a very long time as a lot of consultations would be done via the telephone.
Limitations:	Since in the snowball sampling the authors have decided to prefer reaching out to respondents with further qualifications or experience in policy-making which excluded many of the younger veterinarians, therefore 61% of the veterinarians surveyed were male and only 39% female, which does not accurately reflect the current veterinary population in Portugal (70% of veterinarians are female) (Federation of Veterinarians of Europe, 2019). The sample selection did not require first-hand experience with teleconsultation. This might have led to preconceived impressions being expressed rather than true satisfaction. The dates during which the data was collected are not provided in the study, since teleconsultation is a rapidly developing field it might be outdated. The study might have suffered from ‘end aversion’ bias, which is common when respondents are given a scale on which to express their attitude towards a question and they gravitate towards the middle. This is especially common when the scale consists of an odd number of points, thus promoting a neutral answer. (Choi & Pak, 2005).

**Roca & McCarthy (2019):** 

Population:	Pet owners on a popular telemedicine website.
Sample size:	398 owners.
Intervention details:	Pet owners were divided into two groups – those who had a traditional / in-person veterinarian (Group 1 – 298 owners) and those who did not (Group 2 – 93 owners). Group 1 were asked if they would use teleconsultation if their traditional care veterinarian would provide it whilst Group 2 were asked if they would like a referral to a traditional veterinarian. Owners were then asked if a traditional veterinary consult was recommended and if they followed-up after using telemedicine 159 owners who proceeded to follow up were then asked a set of questions.
Study design:	Cross-sectional study.
Outcome studied:	In the follow-up, the following questions were asked: If owners felt better informed or misinformed about their pet's illness. If their traditional veterinarian (agreed / disagreed) with the recommendation from the ‘telemedicine expert’. If owners were ‘able to communicate their concerns and understand their veterinarians’ recommendations more effectively’ after using telemedicine.
Main Findings (relevant to PICO question)	Pet owners were generally satisfied after using teleconsultation services on the website. 231/384 (60%) owners that used the telemedicine website warranted a follow up with a traditional care veterinarian, of which 159/231 (68.8%) followed through 142/159 (89.3%) felt better informed about their pets’ illness after using teleconsultation however, 14/159 (8.8%) felt misinformed about their pets’ illness after. 131/159 (82.4%) of pet owners reported that their veterinarian agreed with the recommendation from the ‘telemedicine expert’. 138/159 (86.8%) of owners were ‘able to communicate their concerns and understand their veterinarians’ recommendations more effectively’ after using teleconsultation.
Limitations:	Survey was limited to one website, hence a bias towards people who are already using teleconsultation and so are more likely to have a positive attitude towards it. Selection bias due to voluntary surveys as owners with a more positive outlook towards teleconsultation could be more likely to complete survey. ‘Telemedicine expert’ included veterinarians but also ‘other highly qualified pet experts’. The study could have suffered from the ‘positive skew’ (a.k.a. positive satisfaction bias) since respondents are more likely to give positive answers when directly asked if they are satisfied (Choi & Pak, 2005).

## Appraisal, application and reflection

Telemedicine is a blanket term which can be generally broken down into three main fields: remote consults (veterinarian to client); referrals (veterinarian-to-veterinarian); mobile health (Teller & Moberly, 2020). In this study the authors have investigated remote consultations (veterinarian to client) for it to be offered as a permanent service and not a reaction to COVID-19 pandemic, during which it became more widespread (Dubin et al., 2021). This can only be encouraged if satisfaction levels of clients and clinicians need to be at least similar or equal to those of in-person consultations. If the evidence base yields positive, the benefits of teleconsultations are immense, for example for those in remote locations where veterinary expertise is very hard to reach or in cases of emergency where the speed of veterinary advice is vital.

Eight papers with varying levels of evidence were critically appraised. However, only one was a randomised controlled clinical trial, making the overall level of evidence weak. This was the study by Bishop et al. (2018) which assessed client and clinician satisfaction when comparing in-person to teleconsultation (videoconference) postoperative recheck examinations. Clients were more satisfied with the teleconsultation in terms of perceiving the veterinarians’ ability to assess their dog, convenience, and their dogs’ being less afraid or nervous. However, the difference was not statistically significant. Nevertheless, all or the majority of clients were comfortable in using the teleconsultation technology provided, were satisfied with the visual and sound quality, and agreed that their dog was more comfortable getting examined remotely.

The six cross-sectional studies provided weak evidence regarding client and clinician levels with regards to veterinary teleconsultation. Three papers (Bishop et al., 2021; Dubin et al., 2021; and Magalhães-Sant’Ana et al., 2020) assessed levels of clinician satisfaction, two papers (Grisel, 2017; and Roca & McCarthy, 2019) assessed client satisfaction, and Butler et al. (2021) looked at both clinician and client satisfaction. All six papers have a general consensus that when teleconsultation is feasible, levels of satisfaction can be similar (or even higher) to that of traditional or in-person consultations, however there are caveats and exceptions to this.

In the equine industry, Butler et al. (2021) found that both veterinarian and trainer found it useful to triage racehorses by exchanging images and videos. Problems were found with some trainers being reluctant to be billed and it was agreed that teleconsultation is not a replacement to traditional veterinary care. Grisel (2017) found that 59/83 (71%) of horse owners and trainers already used telemedical evaluation for lameness at least once, with 51/59 (86%) being satisfied with the review. 65/83 (78%) of clients prefer if veterinarians offered telemedical review as a service and almost all 81/83 (97.6%) would pay for this service if it was provided. Although not an issue of the method, this paper focused only on lameness consults. While lameness is a very important part of equine medicine, these results should be extrapolated with care as they are based only on one specific presentation that lends itself nicely to remote consultations. Only one of the six papers briefly touched on farm animal practice, which highlighted that teleconsultation was already done for a very long time as often consultations would be done via a telephone call (Magalhães-Sant’Ana et al., 2020).

In small animal practice, teleconsultation may result in both satisfaction for clinicians and clients alike. Most veterinarians (98/135 [72.6%]) spend the same or less amount of time, and have little to no difficulty in adapting to videoconferencing (86/130 [66.2%]) (Bishop et al., 2021).  A study on Portuguese veterinarians found that half of them (21/41 [51%]) agreed that remote consultations can replace consults in certain areas, and the majority (25/41 [61%]) agreed that it would improve animal healthcare (Magalhães-Sant’Ana et al., 2020). Dubin et al. (2021) listed positive client feedback, improved COVID-19 safety, and increased efficiency, as benefits of teleconsultation. In a survey of nearly 400 users of a teleconsultation website, 142/159 (89.3%) of users (pet owners) felt better informed after using their teleconsultation service, and 131/159 (82.4%) of veterinarians agreed with the recommendation from the telemedicine expert (veterinarian or non-veterinarian), implying a good level of both client and clinician satisfaction. Majority of owners were also able to communicate their concerns and understand their veterinarians’ recommendations more effectively (Roca & McCarthy, 2019).

In general, clinicians are found to have more concerns compared to clients when it comes to teleconsultation.

In one study, 103/135 (76%) veterinarians reported less financial compensation and all 135 found it more difficult to foster a good client relationship and to convey information (Bishop et al., 2021). 28/41 (68%) veterinarians agree that whilst teleconsultation is useful, it should be preceded by a physical exam (Magalhães-Sant’Ana et al., 2020). Nevertheless, despite the weak nature of the evidence, the current review suggests that there are areas in which teleconsultation would be appropriate such as post-surgical care (Bishop et al., 2019; 2021), nutrition (Bishop et al., 2021), emergency medicine and triage (Magalhães-Sant’Ana et al., 2020; Donham & Wickett, 2018; and Dubin et al., 2021), behavioural medicine (Magalhães-Sant’Ana et al., 2020), and dermatology (Dubin et al., 2021). However, more studies specifically looking at satisfaction are required to substantiate these claims further.

There is one more paper (Donham & Wickett, 2018) that supported the positive outlook of veterinary teleconsultation, despite not directly assessing satisfaction levels. Donham & Wickett (2018) produced a case report that demonstrated the benefits of teleconsultation in remote locations where veterinary care is not available, showing that it can be vital in emergency cases. Evidence compiled in the systematic literature review found that teleconsulting between the veterinarian and client was generally favoured by both parties (Teller & Moberly, 2020). It described various benefits of teleconsultation including ‘improving access, convenience, enhanced veterinary-to-client bond, reduced workload on front office staff, and is a better option than consulting the internet’. Arguments for its use is especially strong for that of remote locations, or in consults such as behavioural medicine when specialists are limited.

The main issue with the body of evidence reviewed is the subjectivity of measuring satisfaction. The lack of clear definitions of subjectivity and approaches of measuring it, led to each study using different methods which varied greatly (e.g., the outcomes of a semi-structured interview (such as Butler et al., 2021) are very different to a semi-quantified questionnaire (such as Magalhães-Sant’Ana et al., 2020). Even within one format such as a questionnaire, different authors asked differently phrased questions, and used subjective non-standardised terms like ‘excellent’, ‘good’, ‘poor’ to calculate semi-quantitative scores. For example, both Roca & McCarthy (2015) and Bishop et al. (2021) looked at owner satisfaction, but their questions were geared towards their sample populations concerns and were phrased slightly differently. All this makes interpretation and comparison of the results difficult, hence overarching conclusions are unreliable. Furthermore, while some studies measured satisfaction of clients or clinicians directly (Grisel, 2017; and Roca & McCarthy, 2019), some others did not. Some of them set out to primarily investigate the benefits and complications associated with remote consultations instead. For example, in Dubin et al., 2021 most survey questions explored the positive and negative aspects of teleconsultations and most appropriate clinical scenarios to apply teleconsultations, and while providing a very meaningful insight did not measure satisfaction directly.

Another issue found across the studies evaluated was the relatively small sample size e.g., Magalhães-Sant’Ana et al’s. (2020) study used a sample size of 41 to represent the whole population of veterinarians in Portugal. In addition, since participation in all of the studies was voluntary, those with strong feelings towards the subject were more likely to respond to the surveys and questionnaires. It is also likely to be the cause for all client satisfaction data being strongly positive, since only a very small proportion of the total population of clients was sampled. As opposed to the veterinarians, which are a much smaller population and a relatively more representative sampling might have occurred. Therefore, the field requires further research with both larger sample sizes and study designs aimed at measuring satisfaction directly.

Randomised controlled study is the ideal design to measure satisfaction directly, since blinding is impossible in this context. The randomised controlled study carried out by Bishop et al. in 2018 can be used as a model of what could be done, yet while that study focused only on post-surgical checks, similar studies could be done for other types of consultations to see if the satisfaction levels found by Bishop et al. (2018) are validated. This is important, as it was found after reviewing the studies, that not all types of consultations suit remote consults equally, with some being even better than in-person (e.g., behavioural) (Magalhães-Sant’Ana et al., 2020; and Teller & Moberly, 2020).

Other important areas of further study are the growing fields of veterinarian-to-veterinarian consulting and mobile health, which were both outside of the scope of this investigation, yet they constitute a large part of telemedicine and are playing an increasing role in the modern veterinary practice. Furthermore, despite farm veterinary medicine having employed teleconsultation (via telephone consultations) for a much longer time than other fields, to the authors’ knowledge, no literature is available in that field.

While the evidence is, overall, weak, the studies appraised demonstrated that teleconsultation may lead to similar levels of client and clinician satisfaction when compared to in-person consultations but only in very limited circumstances as discussed previously. However, due to varying approaches of measuring satisfaction, each study assessed slightly different aspects of satisfaction. Therefore, there is no conclusive consensus on the totality of the components of satisfaction. This finding is in line with what was found in a review of human telehealth where it was concluded that ‘in most cases, telehealth was equivalent to in-person care, and in some areas, like telerehabilitation and telenutrition, it was better’ (Teller & Moberly, 2020). However, with veterinary teleconsultation being an emergent subject, not enough evidence is available to substantiate this conclusion and further studies, ideally randomised controlled trials, are needed. The current applicability of veterinary teleconsultation was still found to be very limited to certain select scenarios in which it is most appropriate. Relating back to the question raised in the clinical scenario, the existing evidence suggests that teleconsultation can provide similar levels of satisfaction compared to in-person consults in certain circumstances, however the clinician must take into account the number of caveats mentioned previously. When scheduling consults, it is important to select which cases lend themselves best to this format.

## Methodology Section

**Search Strategy:** 

Databases searched and dates covered:	CAB Abstracts accessed via CAB Direct platform (1973–December 2021) PubMed NCBI (1988–December 2021) Web of Science (1900–December 2021)
Search strategy:	CAB Abstracts: (owner* OR client* OR trainer* OR farm* OR veterinar* OR clinician*) (telemedicine* OR teleconsult* OR telehealth* OR telecommunication* OR telediagno* OR telemetr* OR televet* OR teletriage* OR “e-health" OR “virtual consult*” OR “remote consult*” OR “online consult*” OR “video conferenc*”) (“in-person*” OR “face-to-face*” OR “real life*” OR “in person*” OR “real-life*” OR normal* OR “in-clinic*” OR clinic* OR traditional* OR office* OR consult*) (satisfaction* OR impact* OR perception* OR opinion* OR effective* OR efficien* OR attitude* OR perspective* OR perceive* OR view*) ab:(“Veterinar*”) 1 and 2 and 3 and 4 and 5PubMed: (((owner* OR client* OR trainer* OR farm* OR veterinar* OR clinician*)) AND (telemedicine* OR teleconsult* OR telehealth* OR telecommunication* OR telepathology* OR telediagno* OR telemetr* OR televet* OR teletriage* OR “e-health” OR “virtual consult*” OR “remote consult*” OR “online consult*” OR “video conferenc*”)) AND ("in-person*" OR "face-to-face*" OR "real life*" OR "in person*" OR "real-life*" OR normal* OR "in-clinic*" OR clinic* OR traditional* OR office* OR consult*)) AND (satisfaction* OR impact* OR perception* OR opinion* OR effective* OR efficien* OR attitude* OR perspective* OR perceive* OR view*) AND ("Veterinar*"[title/abstract]) Web of Science: ((((TS=(owner* OR client* OR trainer* OR farm* OR veterinar* OR clinician*)) AND TS=(telemedicine* OR teleconsult* OR telehealth* OR telecommunication* OR teleradiolog* OR telepatholog* OR telediagno* OR telemetr* OR televet* OR teletriage* OR “e-health” OR “virtual consult*” OR “remote consult*” OR “online consult*” OR “video conferenc*”)) AND TS=(“in-person*” OR “face-to-face*” OR “real life*” OR “in person*” OR “real-life*” OR normal* OR “in-clinic*” OR clinic* OR traditional* OR office* OR consult*)) AND TS=(satisfaction* OR impact* OR perception* OR opinion* OR effective* OR efficien* OR attitude* OR perspective* OR perceive* OR view*)) AND AB=(Veterinar*)
Dates searches performed:	22 Dec 2021

**Exclusion / Inclusion Criteria:** 

Exclusion:	Not in English language, not related to PICO question, not related to first opinion veterinary consultations, not related to satisfaction levels, not a primary source.
Inclusion:	Related to PICO question, related to first opinion veterinary consultations, related to satisfaction levels.

**Search Outcome:** 

Database	Number of results	Excluded – Not related to first opinion veterinary consultations	Excluded – Not related to satisfaction levels	Excluded – Not a primary source	Total relevant papers
CAB Abstracts	21	11	3	1	6
PubMed	32	24	0	1	7
Web of Science	25	18	1	0	6
Total relevant papers when duplicates removed					8

## Conflict of interest

The authors declare no conflicts of interest.
